# 2008 ACMP Meeting ‐ Young Investigators' Symposium Abstracts

**DOI:** 10.1120/jacmp.v9i3.2881

**Published:** 2008-06-23

**MO‐D‐MET‐01**

## A phantom‐based study for recreating a 4D dynamic dose to account for organ motion in radiotherapy

T Roland

Y Liu

N Papanikolaou

Cancer Treatment and Research Center at The University of Texas Health Science Center, San Antonio, TX

## Purpose

To recreate a four‐dimensional (4D) dose distribution from the planned dose based on one phase of a 4D computed tomography (CT) image and compare with results from 4D dose reconstructed from multiple phases of the 4D CT image and phantom measurements.

## Materials and Methods

The transformation assumes a one‐dimensional sinusoidal tumor motion with amplitude (A) in the inferior‐superior (y) direction. Let ${\;}^{3D}D_{r}(x,\;y,\;z)$ be the three‐dimensional (3D) static dose distribution based on the r^th^ phase and ${\;}^{4D}D(x,\;y,\;z)$ be the corresponding 4D dynamic dose distribution. Then assuming phantom symmetry in the tumor direction,

(1) $${\;}^{4D}D(x,y,z)=\sum_{i=1}^{N}W_{i}\cdot^{3D}D_{r}[x,(y+A\sin\theta_{i}),z]$$

we used a commercial radiation treatment planning system (Pinnacle, Philips Medical Systems, Andover, MA) to plan on a respiratory motion phantom. Eight plans were developed on 8 phases of the tumor cycle. The optimized dose distribution from one phase was then used in (1) to compute the 4D dose distribution for a planar film assumed to move with the tumor center. A weighted average of all the plans constituted the multi‐phase dynamic dose. The plan was delivered on a linear accelerator and measurements were made using radiochromic film.

### Results and Conclusions

Using gamma index analysis, the number of pixels exceeding a gamma index of 1 was 7% and 0% for the single‐phase dose versus the measurement and the reconstructed multi‐phase dose respectively (dose difference tolerance 5%, distance‐to‐agreement tolerance‐5 mm, dose gradient threshold 30%/cm). The close agreement shows that with knowledge of the tumor trajectory and by assuming symmetry in the tumor trajectory direction, a 4D dynamic dose distribution can be recreated fairly well based on a 3D static dose using a single set of CT images.

**MO‐D‐MET‐02**

## Improving the utility of in‐room video camera systems for continuous surveillance of patient motion during radiation treatment

C Kut

C Chen

J Wong

R Taylor

Johns Hopkins University, Baltimore, MD

## Purpose

To improve the utility of in‐room video‐based systems for continuous quantitative monitoring of patient motion, independent of environmental changes in lighting and geometry.

## Methods and Materials

A video‐based tracking system was developed using a webcam with 600×450 pixels and Microsoft Visual C++. The camera was mounted at the end of the treatment couch to constantly view a specified region on the patient's torso of 50×30 cm. Small high contrast “sticky” markers are placed within the viewing region for easy detection. Environmental disturbances are minimized through appropriate real‐time image subtraction and processing techniques. An alarm is activated when the patient movement is deemed out of tolerance. For validation, a lead ball phantom and a flat “sticky” marker of 1 cm diameter were used to determine detectable target displacements. Each tracking session was automatically recorded for further data analysis.

## Results

Target displacements of (2, 2, 5) mm for the lead ball phantom and (4,4,7) mm for the flat phantom are readily detected in the lateral, superior‐inferior and vertical directions, respectively. Most importantly, the system detects these changes in the presence of environmental disturbances, which include large changes in room lighting, and couch and gantry positions.

## Conclusion

Our system provides an effective approach to track and monitor patient position after highly accurate setup using cone‐beam CT, for example. It is considerably lower in cost relative to existing commercial tracking systems. With further refinement, the system can be adapted for routine clinical use that is superior to present in‐room video‐monitoring systems.

**MO‐D‐MET‐03**

## New generation portal sensor based on thin‐film cadmium telluride for clinical high energy X‐ray imaging

J Kang1^1^

I Parsai^2^

D Shvydka^2^

University of Toledo,^1^ and Radiation Oncology Department,^2^ University of Toledo, Toledo, OH

## Purpose

Currently, most popular electronic portal imaging devices are manufactured from hydrogenated amorphous silicon, a material with low atomic number and electron density, exhibiting low quality images and poor radiation hardness. We propose a new generation portal imager based on thin‐film cadmium telluride (CdTe), offering device improvement in both imaging and dosimetric applications. We evaluate material/thickness combinations for the proposed device and estimate its output under typical radiation treatment conditions.

## Methods and Materials

Due to very small thickness (in a range of 100 microns), the sensor has to be combined with a metal plate facilitating conversion of high‐energy photons to charge carriers directly, maximizing the dose deposited in the sensor layer. We employed Monte Carlo package MCNP5 to model the image detection procedure under a 6 MV photon beam from a linear accelerator (SL25, Elekta, Norcross, GA). Several metals in a broad thickness range were analyzed in conjunction with CdTe to find the optimum combinations. We also evaluated the effect of CdTe layer thickness on frequency‐dependent detector quantum efficiency DQE(f) of the device.

## Results

Based on calculations of DQE(f) we proved the CdTe‐based detector system to have higher performance than those using amorphous silicon or selenium. We established the optimal material/thickness combinations for thin‐film CdTe/metal plate detector and found that resultant charge carrier generation leads to the voltage output of 0.2–0.3 volts. We confirmed this voltage output range with our measurements.

## Conclusion

We found the thin‐film CdTe‐based detector is well suited for imaging with high energy X‐rays used in clinical radiation therapy.

**MO‐D‐MET‐04**

## Normalized Bragg peak curves for various proton incident energies in water phantom: a simulation with GEANT4 Monte Carlo code

Y Chen

S Ahmad

Oklahoma University Health Sciences Center, Oklahoma City, OK

## Purpose

To simulate proton interactions (energy from 60 to 250 MeV with intervals of 10 MeV) in water using GEANT4 (version 4.8.3) Monte Carlo code that utilizes electromagnetic and hadronic physics to determine accurately the proton ranges from the Bragg peak.

## Methods and Materials

A cylindrical water phantom $\left({\text{length}=100\;\text{cm},\;\text{diameter}=30\;\text{cm},\;\text{density}=1\;g/\text{cm}}^{3}\right)$ consisting of 1000 circular sensitive detector discs (diameter=2 cm, thickness=1 mm) was used. The simulations were carried out each by 2 million incident protons for each energy with pencil beam; and for five energies (60, 100, 150, 200 and 250 MeV) with 5×5 cm beams. The low‐energy electromagnetic process (protons 1 keV, electrons and photons 250 eV) was used to simulate ionization around the Bragg peak. Range cut is lowered from the default value of 1 mm to 15 um to improve the accuracy of simulation. The hadronic process includes low energy elastic and inelastic scattering that consists of a pre‐compound nuclear interaction below 170 MeV, and a Bertini cascade model for energies above 150 MeV.

## Results

Our simulated normalized Bragg peak curves when compared with the continuous slowing down approximation data from NIST are in excellent agreement. The proton beam penetrates further with increasing energy broadening the Bragg peak with proximal shoulder dose increase from 20% to 40%.

## Conclusions

We have demonstrated that the GEANT4 tool kit has the ability to simulate radiation therapy proton beams and will be used for proton facility design in our institution.

**MO‐D‐MET‐05**

## Monte Carlo simulation of depth dose in water phantom for multi‐energy protons

Y Liu,^1,2^

B Guo^2^

N Papanikolaou^1,2^

University of Texas, Cancer Therapy and Research Center,^1^ and University of Texas Health Science Center,^2^ San Antonio, TX

## Purpose

The purpose is to simulate depth dose spatial distributions in water phantom for multi‐energy therapeutic proton beams using Monte Carlo simulation.

## Methods and Materials

The depth dose for a multi‐energy proton beam was first calculated by mathematical linear approximation, based on pre‐calculated depth dose curves of monochromic proton beams from a wide range of energies. The result was compared to Monte Carlo simulation of three‐dimensional (3D) dose distributions generated by the multi‐energy proton beams in water. The isodose lines and the dose volume histogram to the target were acquired by superimposing the 3D dose matrix to the water phantom's computed tomography (CT) scan.

## Results

The mathematical linear approximation and the Monte Carlo calculation for depth dose distribution gave very close results (R2=0.9998). The 3D dose distribution shown at the axial, coronal and sagittal planes attested the uniformity of dose distribution for proton beam with appropriate spectrum setting. The isodose lines and the dose volume histogram showed that for a sphere target, by simply changing the spectrum of protons, a multienergy proton beam can produce a comparable coverage to target and sufficiently sparing the structure surrounding.

## Conclusion

Proton therapy can deliver high uniform dose to target while sparing dose to the surrounding healthy tissue. By varying the energy spectrum, the depth dose peak range can be adjusted according to the target shape. The Monte Carlo method provides a reliable tool for dose calculation for multi‐energy proton beams. The Monte Carlo calculation provides an accurate 3D depth dose distribution and offers basic and benchmarking data.

## Comprehensive evaluation of Radiation Oncology Information Systems (ROIS)

L Fong

M Herman

Mayo Clinic, Rochester, MN

Radiation Oncology Information Systems (ROIS) have become the bridge between the management of information, technological innovations, patient treatment and high quality patient care. There has been considerable interest from the radiation oncology community in identifying what ROIS best fits a given clinical practice (specific needs and goals). An objective tool to analyze site‐specific clinical information flow and infrastructure, and measure its level of integration with a given ROIS is presented. The proposed methodology is based on identifying and understanding the components of a modern radiation oncology practice. These component objects were classified into the following: Clinical Processes, Information Management and Technological Innovations Integration. Comprehensive IS/IT infrastructure and clinical process maps were generated by a team of experts, representing clinical constituents. These maps served as the basis for evaluating connectivity and process flow and to guide the development of a quantitative survey with user's feedback‐based qualitative information to assess the performance of a given ROIS. Clinical tasks and processes were 1) ranked according to importance for patient care and 2) scored by the team members for performance. The combination of experiential feedback with survey data provided both global and detailed suggestions to improve ROIS integration and site‐specific processes. The hierarchy and importance of various characteristics are customizable to a given clinical practice and thus allow the tool's broad applicability. Proper assessment of information flow and matching of an ROIS will provide a more efficient and more effective care delivery setting.

Conflict of interest: Research supported in part by Varian Medical Systems

